# iPSC screening for drug repurposing identifies anti‐RNA virus agents modulating host cell susceptibility

**DOI:** 10.1002/2211-5463.13153

**Published:** 2021-04-06

**Authors:** Keiko Imamura, Yasuteru Sakurai, Takako Enami, Ran Shibukawa, Yohei Nishi, Akira Ohta, Tsugumine Shu, Jitsutaro Kawaguchi, Sayaka Okada, Thomas Hoenen, Jiro Yasuda, Haruhisa Inoue

**Affiliations:** ^1^ Center for iPS Cell Research and Application (CiRA) Kyoto University Japan; ^2^ iPSC‐Based Drug Discovery and Development Team RIKEN BioResource Research Center (BRC) Kyoto Japan; ^3^ Medical‐risk Avoidance based on iPS Cells Team RIKEN Center for Advanced Intelligence Project (AIP) Kyoto Japan; ^4^ Department of Emerging Infectious Diseases, Institute of Tropical Medicine (NEKKEN) Nagasaki University Japan; ^5^ National Research Center for the Control and Prevention of Infectious Diseases (CCPID) Nagasaki University Japan; ^6^ R&D Center ID Pharma Co., Ltd. Tsukuba Japan; ^7^ Institute of Molecular Virology and Cell Biology Friedrich‐Loeffler‐Institut Greifswald ‐ Insel Riems Germany

**Keywords:** Ebola virus, human iPSC, PPARγ, SARS‐CoV‐2, Sendai virus, SERMs

## Abstract

Human pathogenic RNA viruses are threats to public health because they are prone to escaping the human immune system through mutations of genomic RNA, thereby causing local outbreaks and global pandemics of emerging or re‐emerging viral diseases. While specific therapeutics and vaccines are being developed, a broad‐spectrum therapeutic agent for RNA viruses would be beneficial for targeting newly emerging and mutated RNA viruses. In this study, we conducted a screen of repurposed drugs using Sendai virus (an RNA virus of the family *Paramyxoviridae*), with human‐induced pluripotent stem cells (iPSCs) to explore existing drugs that may present anti‐RNA viral activity. Selected hit compounds were evaluated for their efficacy against two important human pathogens: Ebola virus (EBOV) using Huh7 cells and severe acute respiratory syndrome coronavirus 2 (SARS‐CoV‐2) using Vero E6 cells. Selective estrogen receptor modulators (SERMs), including raloxifene, exhibited antiviral activities against EBOV and SARS‐CoV‐2. Pioglitazone, a PPARγ agonist, also exhibited antiviral activities against SARS‐CoV‐2, and both raloxifene and pioglitazone presented a synergistic antiviral effect. Finally, we demonstrated that SERMs blocked entry steps of SARS‐CoV‐2 into host cells. These findings suggest that the identified FDA‐approved drugs can modulate host cell susceptibility against RNA viruses.

## Graphical abstract

FDA‐approved drugs, identified by iPSC screening with Sendai virus, exhibited inhibitory effects on the infection of Huh7 cells by Ebola virus and of Vero E6 cells by SARS‐CoV‐2. These drugs, with efficacy in different combinations of RNA viruses and cells, may have therapeutic potential for RNA virus infections, including newly emerging and mutated viruses, by modulating host cell susceptibility.

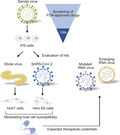

**Abbreviations**
DAPI: 4’6‐diamidino‐2‐phenylindole
EBOV: Ebola virus
EGFP: enhanced green fluorescent protein
iPSCs: induced pluripotent stem cells
MOI: multiplicity of infection
SARS‐CoV‐2: severe acute respiratory syndrome coronavirus 2
SERMs: selective estrogen receptor modulators
VSV: vesicular stomatitis virus
ZIP: zero interaction potency

The emergence of severe acute respiratory syndrome coronavirus 2 (SARS‐CoV‐2) has caused a pandemic and posed a threat to public health due to its efficient human‐to‐human transmission and occasional display of high pathogenicity [1, 2]. SARS‐CoV‐2 belongs to the *Coronaviridae* family, which is an enveloped virus possessing a single‐stranded, positive‐sense RNA genome [3]. Other emerging viral diseases known as SARS and Middle East respiratory syndrome are also caused by members of *Coronaviridae*, whereas highly lethal Ebola virus (EBOV) disease and Marburg virus disease are caused by members of the *Filoviridae* family, which are also enveloped viruses with single‐stranded, negative‐sense RNA genomes. Besides coronaviruses and filoviruses, many other RNA viruses also present a threat of emerging and re‐emerging infectious diseases, and therefore, it is of great importance to find a therapeutic agent for RNA virus‐related diseases.

RNA viruses are the most common cause of emerging and re‐emerging infectious diseases due to their high mutation rate [4]. RNA viruses in different families exhibit unique particle morphology with highly diverse gene compositions, presenting a biological diversity with different pathogenicities in different hosts. Although specific therapeutics and vaccines are being developed, a common therapeutic agent for a broad range of RNA viruses may be beneficial, since the agent could be a good candidate as an antiviral drug against newly emerging RNA viruses such as the current pandemic SARS‐CoV‐2. New approaches to identifying a broad‐spectrum antiviral target shared by different viruses are expected.

In the current study, to find a repurposed drug inhibiting multiple RNA viruses, as a first step, we used Sendai virus (SeV) and human‐induced pluripotent stem cells (iPSCs) to screen FDA‐approved drug libraries. SeV is a single‐stranded, negative‐strand RNA virus belonging to the genus *Respirovirus* of the *Paramyxovirinae* subfamily of the *Paramyxoviridae* family. SeV is a mouse parainfluenza virus type 1 discovered in Sendai, Japan, and it naturally replicates in respiratory mucosa [5, 6]. A recombinant attenuated form has been produced by genetic engineering [7], and SeV carrying the target gene is used for human gene therapy [8, 9, 10]. Human iPSCs have been widely used for disease modeling and drug discovery [11, 12], and even undifferentiated iPSCs themselves are useful for compound screening [13], providing the advantage of harboring human genes with normal karyotypes and infinite self‐renewal ability.

In this study, we established a compound screening system with human iPSCs to measure viral infectivity by detecting enhanced green fluorescent protein (EGFP) expressed by SeV. The antiviral efficacy of selected hit drugs was further evaluated by EBOV and SARS‐CoV‐2, and FDA‐approved drugs were identified as broad‐spectrum drugs against RNA viruses.

## Materials and methods

### Ethics statements

The generation and use of human iPSCs were approved by the Ethics Committee of Kyoto University. The study methodologies conformed to the standards set by the declaration of Helsinki. Formal written informed consent was obtained from the subject.

### Generation of human iPSCs

Human iPSCs were generated from peripheral blood mononuclear cells using episomal vectors (Sox2, Klf4, Oct3/4, L‐Myc, Lin28, and p53‐shRNA) as reported previously [14] and were cultured by feeder‐free culture system with StemFit (Ajinomoto, Tokyo, Japan). Karyotype analysis of iPSCs was conducted by LSI Medience (Tokyo, Japan).

### Chemicals for screening

For throughput screening, an FDA‐approved drug library from ENZO Life Science (Farmingdale, NY, USA) was used. For RNA quantification assays, all chemicals were purchased from Selleck Chemicals (Houston, TX, USA).

### Compound screening using SeV with human iPSCs

For throughput compound screening, human iPSCs were dissociated to single cells with TrypLE Express (GIBCO, Thermo Fisher Scientific, Waltham, MA, USA) and were disseminated onto iMatrix‐coated 96‐well plates with StemFit containing 10 μm Y‐27632 (Nacalai Tesque, Kyoto Japan). After 24 h, the culture medium was replaced with fresh StemFit containing compounds for 3 h, and then, iPSCs were infected with SeV carrying the EGFP gene. Multiplicity of infection (MOI) was estimated to 1. After 48 h of incubation, cells were washed twice with PBS and then fixed in 4% paraformaldehyde (PFA) for 10 min at room temperature. 4’6‐Diamidino‐2‐phenylindole (DAPI) (Life Technologies, Waltham, MA. USA) was used to label the nuclei. Cell images were acquired with IN CELL Analyzer 6000 (GE Healthcare, Chicago, IL, USA) in the throughput screening and IN CELL Analyzer 2000 (GE Healthcare) in the dose dependency assay, and the number of EGFP‐positive cells was quantified using IN CELL Developer toolbox software 1.92 (GE Healthcare).

### Quantitative RT‐PCR

Human iPSCs were seeded onto iMatrix‐coated 24‐well plates with StemFit (Nacalai Tesque) and infected with SeV. After the cells were washed twice with PBS, the total RNA of the cultured human iPSCs was extracted by miRNeasy Mini kit (QIAGEN, Venlo, Netherlands) following the manufacturer's protocol. Five hundred nanogram of RNA was reverse‐transcribed using ReverTra Ace (TOYOBO, Osaka, Japan). Oligo dT was used for reverse transcription to measure the mRNA levels, while random primers were used for reverse transcription to measure the viral genomic RNA levels. Quantitative PCR analysis was performed using reverse transcription reaction with SYBR Premix Ex TaqⅡ (TAKARA, Kusatsu, Japan) and StepOnePlus (Thermo Fisher Scientific). The primer sets are listed in Table S1.

### Cell lines

293T cells, Huh7 cells, and Vero E6 cells (donated by Ayato Takada, Hokkaido University, Japan) were maintained in Dulbecco's modified Eagle's medium supplemented with 10% FBS and 1% penicillin/streptomycin solution.

### Ebola trVLP production and infection

For evaluating anti‐EBOV activities of compounds, Ebola trVLP expressing a GFP reporter was produced using 293T cells as described previously [15]. Briefly, cells were transfected with pCAGGS expression plasmids encoding EBOV‐NP, EBOV‐VP35, EBOV‐VP30, EBOV‐L, T7‐polymerase, and a trVLP tetracistronic minigenome that encodes EBOV‐GP, EBOV‐VP40, EBOV‐VP24, and a GFP reporter gene by the calcium phosphate method. One day after transfection, the culture supernatant was replaced with fresh medium. After three additional days, the supernatant was collected, clarified by centrifugation at 2000 ***g*** for 15 min, and stored at −80 °C until use. To increase the titers, Ebola trVLP was passaged 2–3 times as follows. 293T cells were transfected with pCAGGS expression plasmids encoding EBOV‐NP, EBOV‐VP35, EBOV‐VP30, EBOV‐L, and a host attachment factor Tim‐1 using the calcium phosphate method. One day after transfection, the cells were infected with produced or passaged trVLP for 1 day, and the culture supernatant was collected after additional 3‐day culture. For evaluating the antiviral activity of compounds, Huh7 cells were transfected with pCAGGS expression plasmids encoding EBOV‐NP, EBOV‐VP35, EBOV‐VP30, and EBOV‐L using the TransIT LT1 transfection reagent (Mirus, Madison, WI, USA). Two days after transfection, the cells were pretreated with each compound at appropriate concentration and infected with the passaged trVLP in the presence of the compound for 2 days. After fixing the infected cells with 10% formalin overnight, they were stained with Hoechst 33342 dye for nuclear staining and then imaged by Cytation 5 imaging plate reader (BioTek Instruments, Winooski, VT, USA) with a 4× lens. Counting of the cell nuclei and infected cells was performed using CellProfiler image analysis software (Broad Institute, MIT, Boston, MA, USA) and a customized analysis pipeline.

### SARS‐CoV‐2 propagation and infection

A JPN/NGS/IA‐1/2020 strain of SARS‐CoV‐2 (accession number: EPI‐ISL‐481251, GISAID), which was isolated from a Japanese patient, was propagated in Vero E6 cells. Culture supernatants were collected 4 days after infection, clarified by centrifugation at 2000 ***g*** for 15 min, and stored at −80 °C until use. To evaluate the antiviral activity of compounds, Vero E6 cells were plated in 96‐well plates and incubated with each compound at appropriate concentration for 1 h. The cells were then challenged with SARS‐CoV‐2 at an MOI of 0.002 and incubated in the presence of compounds at 37 °C. After 2 days, the cells were fixed using 4% PFA overnight. Virus infectivity was determined by immunofluorescence using 0.2% Triton X‐100 for permeabilization, 10% goat serum for blocking, rabbit anti‐SARS‐CoV N antibody as a primary antibody, Alexa Fluor 488 goat anti‐rabbit IgG as a secondary antibody, and Hoechst 33342 dye for nuclear staining. The cells were imaged by Cytation 5 imaging plate reader with a 4× lens. Counting of cell nuclei and infected cells was performed using CellProfiler image analysis software and a customized analysis pipeline. All experiments with replication‐competent SARS‐CoV‐2 were performed in a biosafety level 3 laboratory at Nagasaki University.

### Drug combination analysis

To explore the efficacy of drug combinations, the inhibition rate against viral infection (%) was calculated for every dose combination of raloxifene and remdesivir or pioglitazone using SynergyFinder (https://synergyfinder.fimm.fi/) in comparison with each agent alone. The synergy score for the compound combination was calculated with the zero interaction potency (ZIP) model. Synergy scores near 0 give limited confidence for synergy or antagonism. When the synergy score is (a) < −10, the interaction between two drugs is likely to be antagonistic; (b) from −10 to 10, the interaction between two drugs is likely to be additive; (c) larger than 10, the interaction between two drugs is likely to be synergistic [16, 17].

### Production and infection of pseudotyped VSV

To produce vesicular stomatitis virus (VSV) pseudotyped with SARS‐CoV‐2 spike proteins or SARS‐CoV spike proteins (VSVΔG‐SARS2‐S or VSVΔG‐SARS‐S, respectively), codon‐optimized SARS‐CoV‐2 S gene with 19 a.a. deletion of the C terminus and codon‐optimized SARS‐CoV S gene with 19 a.a. deletion of the C terminus were synthesized and inserted into a pCAGGS expression plasmid using In‐Fusion HD (Clontech, Mountain View, CA, USA). VSVΔG‐SARS2‐S and VSVΔG‐SARS‐S were generated using a recombinant VSV with VSV‐G gene replaced by a firefly luciferase reporter gene (VSVΔG‐VSV‐G) as described below. 293T cells were transfected with a pCAGGS plasmid encoding SARS‐CoV‐2 S gene or SARS‐CoV S gene using the calcium phosphate method. One day after transfection, the cells were infected with VSVΔG‐VSV‐G for 1 h. The supernatant was harvested 1 day after infection, clarified by centrifugation at 2000 ***g*** for 15 min, and stored at −80 °C until use. As a control against contamination of the inoculating virus, the cells were transfected with a pCAGGS plasmid and challenged with VSVΔG‐VSV‐G for 1 h, followed by collection of the culture supernatant 1 day after infection. To evaluate the effects of compounds on pseudotyped VSV infection, Vero E6 cells were plated in 96‐well plates and incubated with each compound at appropriate concentration for 1 h. Then, the cells were challenged with each pseudotyped VSV and incubated in the presence of compounds at 37 °C. After 20 h, the cells were lysed and luciferase activity was measured using a SpectraMax iD5 microplate reader (Molecular Devices, San Jose, CA, USA).

### Statistical analysis

Results were analyzed using one‐way ANOVA followed by Dunnett's *post hoc* test to determine statistical significance. A difference of *P* < 0.05 was considered significant. Analyses were performed using GraphPad Prism software version 8.0 for Windows (GraphPad Software, San Diego, CA, USA).

## Results

### Screening for anti‐RNA virus drugs using SeV with human iPSCs

**Fig. 1:**
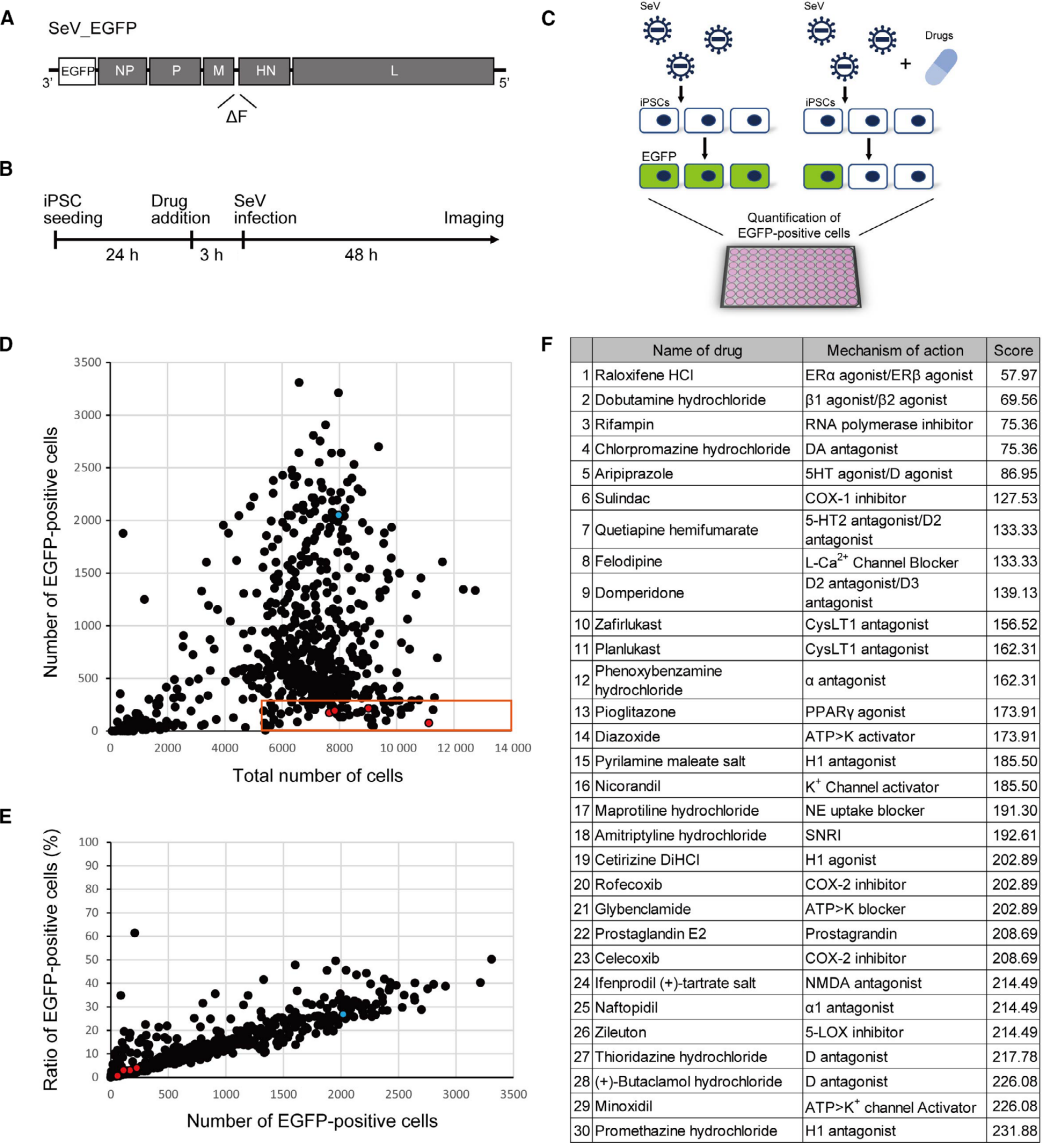
Compound screening using SeV and iPSCs. (A) Construction of SeV. SeV carrying EGFP gene in the 3’ region of viral genomic RNA was constructed after removal of F gene. (B) Timeline of compound screening. (C) Schema of compound screening. (D and E) Result of first screening. Approximately 500 compounds were evaluated. Blue dot shows negative control (DMSO). Red dots show the hit drugs raloxifene, rifampin, pranlukast, zileuton, and pioglitazone. (F) List of hit compounds. The scores reflect the number of EGFP‐positive cells.

Compound screening to identify therapeutic drugs against RNA viruses was conducted using SeV with a human iPSCs‐based assay. The SeV genome contains 3′ leader and 5′ trailer sequences and encodes six structural genes, which are transcribed in the order of nucleocapsid (N), phosphoprotein (P), matrix (M), fusion (F), hemagglutinin–neuraminidase (HN), and large polymerase (L). In this study, SeV carrying EGFP gene in the 3′ region of viral genomic RNA was constructed after removal of the F gene to eliminate the viral ability to produce progeny virions infecting other cells [18] (Fig. 1A). Human iPSCs, which were generated from a healthy subject (Fig. S1A), were infected with SeV, and an assay system was established to measure viral replication by detecting EGFP‐positive iPSCs. We conducted the screening of ~ 500 FDA‐approved existing drugs using this system to detect therapeutic agents against RNA virus‐related diseases. iPSCs, seeded on 96‐well plates, were pretreated with 10‐μm compounds for 3 h, which was followed by SeV infection to detect compounds that modulate host cell susceptibility. Forty‐eight hours after the viral infection, the number of EGFP‐positive cells was quantified, and compounds reducing the number of EGFP‐positive cells were extracted (Fig. 1B,C). Considering the cellular toxicity evaluated by cell count with DAPI staining in the same plate as for the infection assays, compounds showing a great decrease in the number of cells were excluded, and the top 30 compounds with low numbers of EGFP‐positive cells were considered as hits (Fig. 1D–F).

### Inhibition of viral replication by FDA‐approved drugs

**Fig. 2:**
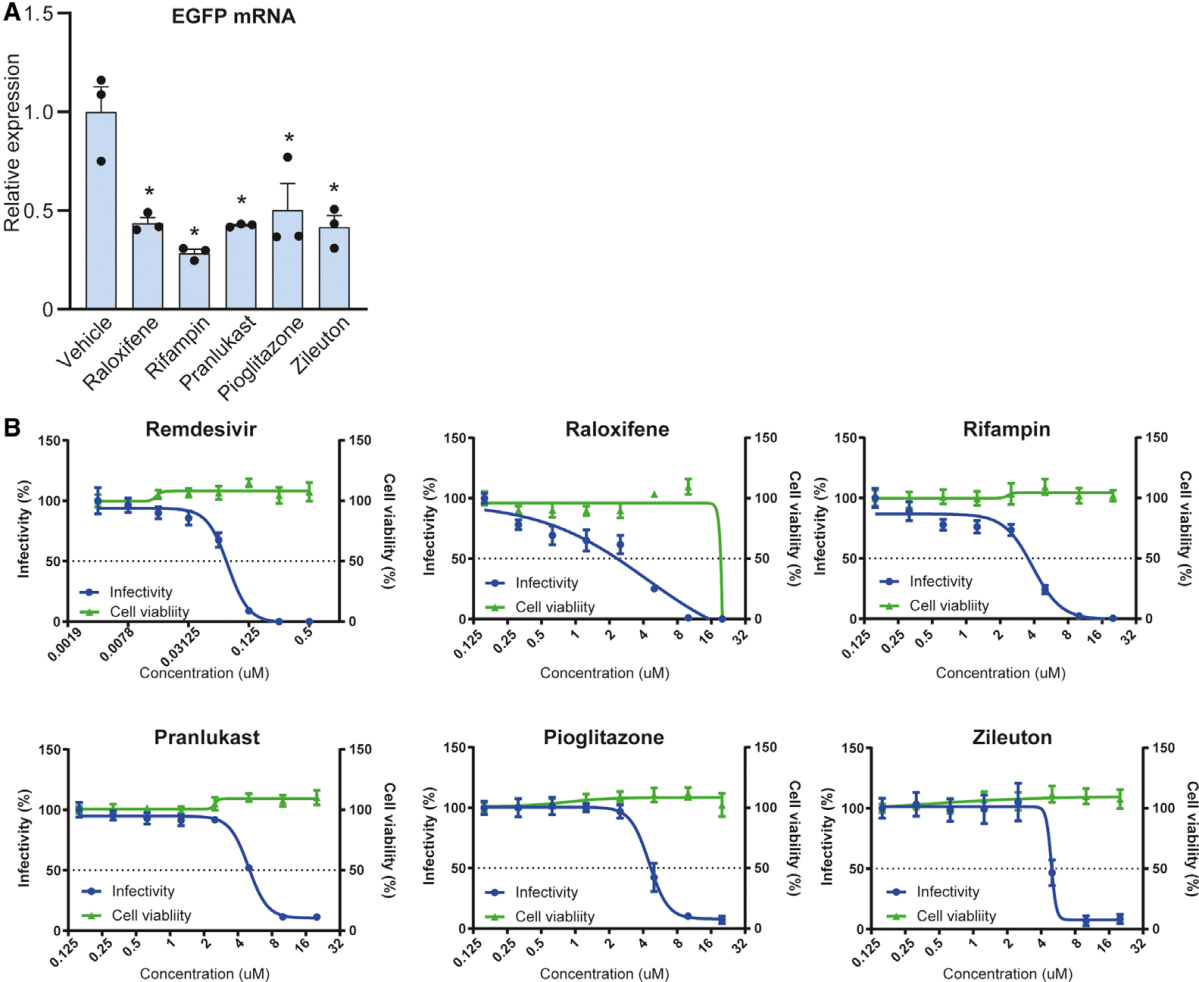
Evaluation of selected compounds using SeV and iPSCs. (A) Quantification of viral mRNA levels. Pre‐treatment of 10 μm raloxifene (Ralo), 10 μm rifampin (Rif), 10 μm pranlukast (Pra), 10 μm zileuton (Zil), or 10 μm pioglitazone (Pio) significantly decreased EGFP mRNA levels (*n* = 3; one‐way ANOVA, *P* < 0.005; Dunnett's *post hoc* test, * *P* < 0.005). Bar graphs represent mean ± SEM. (B) Dose–response analysis of remdesivir and the selected compounds against the infectivity of SeV showing infectivity (blue) and cell survival (green). Data are normalized to mean values for untreated control and presented as mean ± SEM. *n* = 6, biological replicates.

We selected drugs with little effect on cardiovascular circulation and the central nervous system from the hits, and we focused further work on five drugs with different targets: raloxifene, selective estrogen receptor modulator (SERM), rifampin, anti‐tuberculosis, pranlukast, CysLT1 antagonist, zileuton, 5‐LOX inhibitor, pioglitazone, PPARγ agonist for the next analysis. We investigated whether these drugs suppressed viral replication by evaluating mRNA levels. iPSCs, seeded on 24‐well plates, were pretreated with 10‐μm compounds for 3 h, followed by SeV infection. 24 h after viral infection, RNA was extracted from the iPSCs and quantified. Raloxifene, rifampin, pranlukast, zileuton, and pioglitazone significantly inhibited EGFP mRNA production (Fig. 2A), as shown in remdesivir, RNA‐dependent RNA polymerase inhibitor approved by FDA for COVID‐19 (Fig. S1B). This result indicated that the hit compounds inhibited the RNA synthetic activity of the virus or a step in the viral lifecycle upstream of this process.

The dose dependency of these drugs was evaluated against SeV infection. Remdesivir decreased the infectivity of SeV with an IC_50_ value of 0.077 μm, indicating that this assay system is appropriate for assessing RNA virus infection (Fig. 2B). Raloxifene, rifampin, pranlukast, zileuton, and pioglitazone decreased the infectivity of SeV in a dose‐dependent manner with IC_50_ values of 4.3, 3.9, 5.0, 4.5, and 4.9 μm, respectively (Fig. 2B).

### Validation of selected compounds using an EBOV lifecycle modeling system

**Fig. 3:**
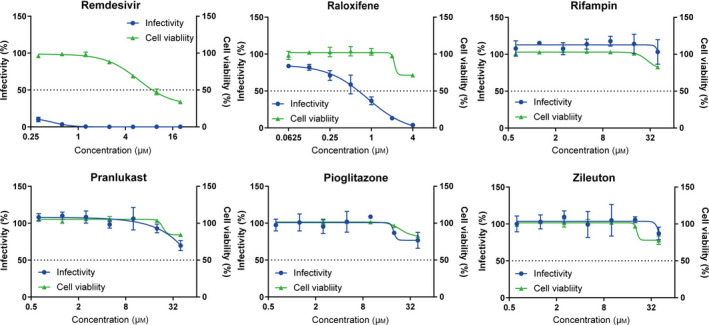
Evaluation of selected compounds using an Ebola trVLP system with Huh7 cells. Dose–response analysis of remdesivir and the selected compounds against the infectivity of Ebola trVLP showing infectivity (blue) and cell survival (green). Raloxifene presented an inhibitory effect against Ebola trVLP infection. Rifampin, pranlukast, pioglitazone, and zileuton did not show significant effects. Data are normalized to mean values for untreated control and presented as mean ± SEM. *n* = 3, biological replicates.

In order to assess the antiviral activity of the selected compounds against human pathogens, they were evaluated for anti‐EBOV activity using a transcription‐ and replication‐competent virus‐like particle (trVLP) system, which does not require a biosafety level 4 laboratory, unlike wild‐type virus [15]. Huh7 cells, derived from a human liver, were infected with Ebola trVLP expressing a GFP reporter, followed by quantification of the number of infected cells as well as determination of cell viability. Remdesivir, a nucleotide analog developed for the treatment of EBOV disease [19], potently inhibited Ebola trVLP infection with an IC_50_ value of 0.12 μm, showing the adequacy of our experimental system (Fig. 3). This was followed by evaluation of the efficacies of raloxifene, rifampin, pranlukast, zileuton, and pioglitazone against the infectivity of Ebola trVLP. We found that raloxifene decreased the infectivity of Ebola trVLP in a dose‐dependent manner with an IC_50_ value of 0.88 μm (Fig. 3). There was a significant divergence between antiviral activity and cellular toxicity, indicating a specific antiviral effect. Rifampin, pranlukast, zileuton, and pioglitazone did not show significant antiviral activities (Fig. 3).

### Validation of selected compounds using SARS‐CoV‐2

**Fig. 4:**
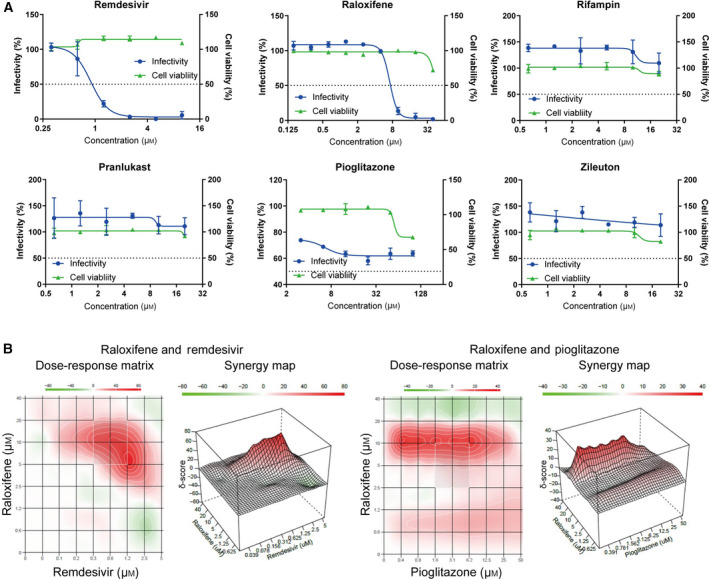
Evaluation of selected compounds using SARS‐CoV‐2 with Vero E6 cells. (A) Dose–response analysis of remdesivir and hit compounds against the infectivity of SARS‐CoV‐2 showing infectivity (blue) and cell survival (green). Raloxifene demonstrated inhibitory effect against SARS‐CoV‐2 viral infection. Pioglitazone showed efficacy at high concentrations. Rifampin, pranlukast, and zileuton did not show significant effects. Data are normalized to mean values for untreated control and presented as mean ± SEM. *n* = 3, biological replicates. (B) Combination effects of selected antiviral compounds. Dose–response matrices and 3‐D drug synergy map plotting synergy scores across all tested compound concentrations are presented. Synergistic effect: δ > 10; additive effect: −10 < δ < 10; antagonistic effect: δ < 10. The combination of raloxifene and remdesivir exhibited a synergistic antiviral effect across specific concentrations. Pioglitazone also showed a weak synergistic effect with raloxifene.

Next, the compounds selected in the SeV screen were evaluated by the use of SARS‐CoV‐2. Vero E6 cells were infected with wild‐type SARS‐CoV‐2, which was followed by quantification of the number of infected cells as well as determination of cell viability using an immunofluorescence technique. For validation of the assay, remdesivir, approved by FDA for COVID‐19, decreased the infectivity of SARS‐CoV‐2 with an IC_50_ value of 0.89 μm (Fig. 4A) as was also indicated in a previous report [20]. Raloxifene, rifampin, pranlukast, zileuton, and pioglitazone were then also evaluated against SARS‐CoV‐2 infection. We found that raloxifene, with an IC_50_ value of 7.1 μm, decreased the infectivity of SARS‐CoV‐2 in a dose‐dependent manner (Fig. 4A). In addition, pioglitazone partially inhibited the virus infection at high concentration (Fig. 4A), while rifampin, pranlukast, and zileuton did not show any significant antiviral activities (Fig. 4A).

We investigated the drug combination effect of raloxifene and remdesivir or pioglitazone using SynergyFinder [16, 17] and found that raloxifene and remdesivir showed a synergistic antiviral effect across specific concentrations, but no significant antagonistic effect at any tested concentrations (ZIP synergy score 6.91, most synergistic area score 30.09). Raloxifene and pioglitazone also showed a small synergistic antiviral effect across specific concentrations (ZIP synergy score 4.49, most synergistic area score 12.42) (Fig. 4B).

### Effects of SERMs against SARS‐CoV‐2 infectivity

**Fig. 5:**
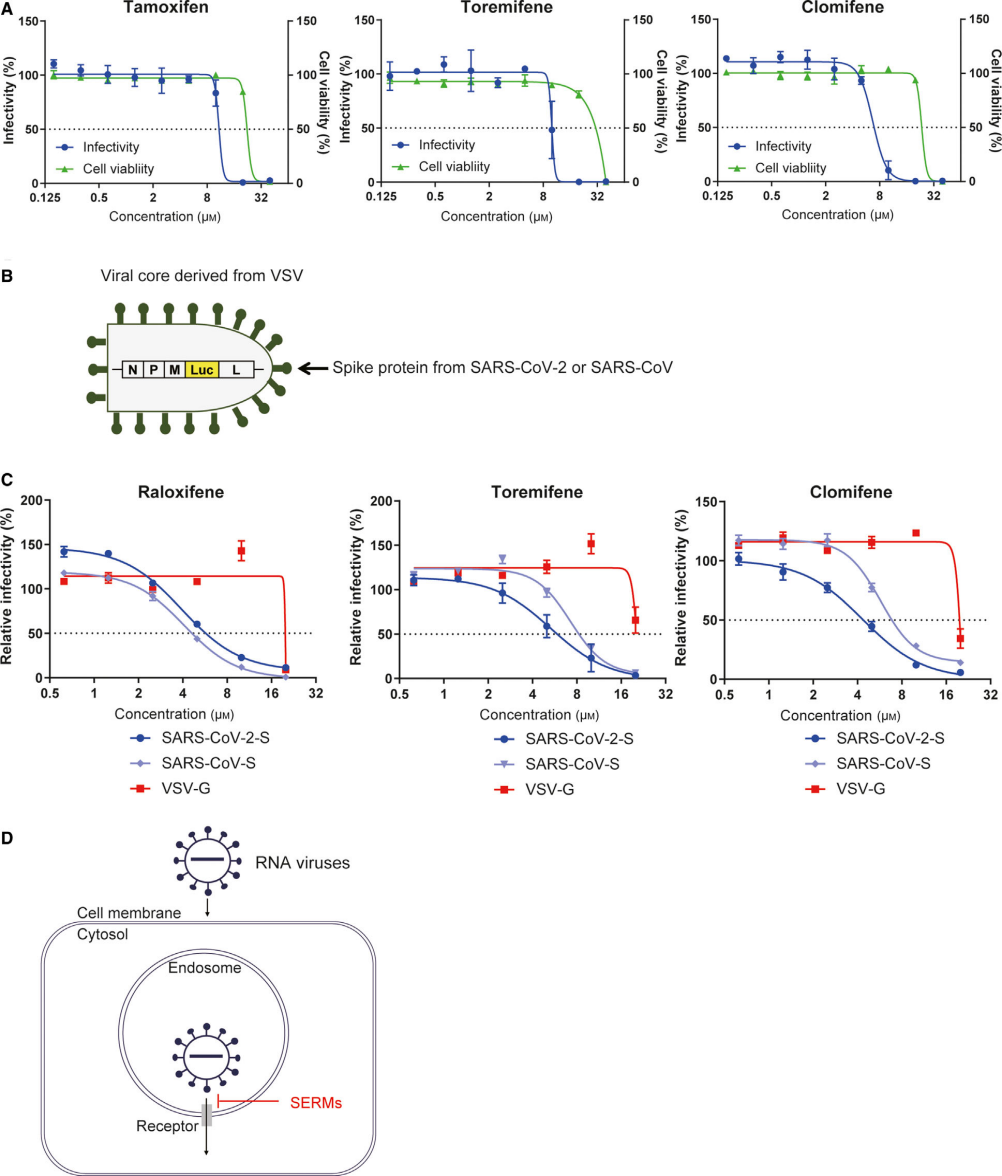
Effect of SERMs on viral entry into host cells. (A) Antiviral activity of SERMs against SARS‐CoV‐2. Dose–response analysis of SERMs is presented. Tamoxifen, toremifene, and clomifene inhibited SAES‐CoV‐2 infection showing infectivity (blue) and cell survival (green). Data are normalized to mean values for untreated control and presented as mean ± SEM. *n* = 3, biological replicates. (B) Schema of pseudotyped VSV. The particle harbors a viral core derived from VSV and spike proteins or glycoproteins from the target virus. G gene is deleted from the original VSV genome, and luciferase reporter gene is inserted. (C) Dose–response analysis of SERMs against viral infectivity of VSVΔG‐SARS2‐S, VSVΔG‐SARS‐S, or VSVΔG‐VSV‐G. Raloxifene, toremifene, and clomifene showed more potent antiviral effects against VSVΔG‐SARS2‐S and VSVΔG‐SARS‐S than VSVΔG‐VSV‐G. Data are normalized to mean values for untreated control and presented as mean ± SEM. *n* = 3, biological replicates. (D) Hypothetical schema of SERMs against RNA virus infection. SERMs may disrupt ion homeostasis inside endosomes containing internalized virus particles and inhibit membrane fusion to release the virus cores into the host cell cytoplasm.

Among our hit compounds, raloxifene showed antiviral activity against both EBOV and SARS‐CoV‐2. It is a member of SERMs, which include several FDA‐approved drugs prescribed for cancer therapy. Using the trVLP system, we confirmed previous reports that multiple members of SERMs potently inhibited EBOV infection (Fig. S2) [21, 22]. According to the efficacy of raloxifene, we focused on the inhibitory effect of SERMs against SARS‐CoV‐2 infection. The effects of tamoxifen, toremifene, and clomifene, SERMs other than raloxifene, were investigated, and these SERMs demonstrated inhibitory effects on SARS‐CoV‐2 infection in Vero E6 cells with IC_50_ values of 10.9, 10.0, and 6.7 μm, respectively (Fig. 5A).

We then analyzed the mechanism of the antiviral effects of these compounds. As previous reports showed that SERMs targeted the host entry step of EBOV [21], we investigated whether SERMs also inhibited the entry of SARS‐CoV‐2 into host cells. The entry of SARS‐CoV‐2 was examined using pseudotype VSV with SARS‐CoV‐2 spike proteins using Vero E6 cells (Fig. 5B). Raloxifene, toremifene, and clomifene inhibited the infection of pseudotype VSV with spike protein (S) of SARS‐CoV‐2 with IC_50_ values of 3.9, 5.3, and 4.4 μm, respectively, while the effects were not observed in VSV with VSV glycoprotein (G) (Fig. 5C). In addition, SERM inhibited the infection of pseudotype VSV with the SARS‐CoV spike protein (S) with IC_50_ values of 4.1, 7.3, 5.8 μm, respectively. These results suggested that SERMs harbored antiviral effects by inhibiting the entry of pathogenic coronaviruses into host cells via viral spike proteins (Fig. 5D).

## Discussion

We conducted compound screening with SeV and human iPSCs for the evaluation of antiviral activity to find a therapeutic candidate against RNA virus‐related diseases. We found that several existing drugs including a SERM, anti‐tuberculosis, anti‐asthmatic, and anti‐diabetic drugs suppressed SeV replication. Among them, SERMs inhibited both EBOV and SARS‐CoV‐2 replication. SERMs exhibited antiviral activities against EBOV and SARS‐CoV‐2, and pioglitazone exhibited antiviral activities against SARS‐CoV‐2. Raloxifene and pioglitazone presented synergistic antiviral effect against SARS‐CoV‐2 in Vero E6 cells.

Potential therapies currently in research and clinical phases for SARS‐CoV‐2 based on drug repurposing include remdesivir developed for EBOV disease [23], favipiravir for influenza and EBOV disease [24], chloroquine for malaria [25], and IL‐6 inhibitors tocilizumab and sarilumab for the treatment of rheumatoid arthritis [26]. In addition, inhibitors of Sigma1 and Sigma2 receptors have been found by screening using cell lines and coronaviruses *in vitro* [27]. Currently, the SARS‐CoV‐2 main protease and the papain‐like protease are also highlighted as drug targets [28, 29, 30, 31, 32].

We found that SERMs inhibited infection by SeV, EBOV, and SARS‐CoV‐2, which all belong to very different families across both negative‐sense and positive‐sense RNA viruses, suggesting that SERMs are one good example for broad‐spectrum RNA virus inhibitors. There have been several reports on the effects of SERMs on RNA viruses. Among them, SERMs have been shown to be effective against SARS‐CoV‐2 [33, 34, 35], EBOV [21], Dengue virus [36], and Zika virus [36]. The mechanism for the antiviral effects of SERMs has been well investigated in the EBOV [21], and it has been reported that SERMs inhibited the viral entry of EBOV into host cells [37]. From this, a similar mechanism has been assumed for other viruses, but it has not been proven. We revealed that SERMs inhibited SARS‐CoV‐2 infection by blocking virus entry into host cells via viral spike proteins. Our findings are supported by a report of prediction by *in silico* network analysis using the transcriptome of human coronaviruses [38]. SERMs have a complex profile of both agonistic and antagonistic modulators of estrogen receptor subtypes that demonstrate effects on the immune system and immune‐mediated inflammatory responses [39]. They present anti‐inflammatory responses and inhibit the release of pro‐inflammatory cytokines [40, 41, 42], and they have also been reported to have anti‐inflammatory effects, suggesting that they may also inhibit severe disease mechanisms related to cytokine storm [43]. Taken together, these findings suggest that SERMs could be a general inhibitor of RNA virus infection.

We also found that pioglitazone harbored an inhibitory effect against SARS‐CoV‐2 infection. Pioglitazone is a selective PPARγ agonist used to treat diabetes. As a recent report identified another PPARγ agonist as inhibiting SARS‐CoV‐2 infection in large compound screening [20], PPARγ seems to regulate virus infection. It has been reported that PPARγ agonist may prove to have a beneficial effect on influenza infection by causing a reduction in inflammatory cytokines [44]. Furthermore, pioglitazone also inhibited viral replication of HIV [45]. The target of pioglitazone is key regulators of inflammation whose activation specifically results in a reduction of inflammatory cytokines such as IL‐6 and INFγ [46]. These pathways may be involved in the inhibition of viral replication, and it is suggested that the pro‐inflammatory pathway of host cells activated by viral infection may promote viral replication in a vicious cycle.

It is important to understand the characteristics of cells used for compound screening. iPSCs expressed only low levels of ACE, and hit compounds identified by iPSC screening were evaluated with Vero E6 cells, African green monkey kidney epithelial cells expressing a high level of the ACE2 receptor. However, they present some limitations by behaving differently from primary airway cells, including a lack of production of interferon when they are infected with certain viruses [47] and a lack of cell surface proteases including TMPRSS2 that activate the entry of SARS‐CoV‐2 from the cell surface into the cell [48]. Furthermore, differences in drug responsiveness between *in vitro* and *in vivo* were reported. The antimalarial compounds chloroquine and hydroxychloroquine could inhibit SARS‐CoV‐2 in Vero E6 cells, but there was no clinical improvement in patients [49]. Thus, biological observations obtained with cell lines would need to be carefully validated in multiple models including primary human airway cells, human iPSC‐derived alveolar cells [50], and/or animal models.

We demonstrated that iPSC screening using SeV identified FDA‐approved drugs as harboring an inhibitory effect on the infection of SeV, EBOV, and SARS‐CoV‐2 by modulating host cell susceptibility against RNA viruses. These drugs may have therapeutic potentials for RNA virus infections universally, and further mechanistic investigations could support a facilitation of their clinical application.

## Conflict of interest

The authors declare no conflict of interest.

## Author contributions

HI conceived the project. KI, YS, JY, and HI designed the experiments. KI, YS, TE, RS, and SO performed the experiments. YN and AO provided compound libraries. TS and JK performed the SeV vector design and construction. TH developed and provided the Ebola trVLP system. KI, YS, TH, JY, and HI wrote the manuscript.

## Supporting information


**Fig. S1.** Karyotype analysis of iPSCs and evaluation of an RNA‐dependent RNA polymerase inhibitor. A. Karyotype analysis of iPSCs. B. Quantification of viral mRNA levels. Pre‐treatment of 1 μm Remdesivir significantly decreased EGFP mRNA levels (*n* = 3; student *t*‐test, * *P < *0.005). Bar graphs represent mean ± SEM.
**Fig. S2.** Antiviral activity of SERMs against Ebola trVLP. Dose‐response analysis of SERMs against the infectivity of Ebola trVLP. Tamoxifene, Toremifene, and Clomifene inhibited Ebola trVLP infection showing infectivity (blue) and cell survival (green). Data are normalized to mean values for untreated control and presented as mean ± SEM. *n* = 3, biological replicates.
**Table S1.** Primer list for qPCR.

## Data Availability

The data that support the findings of this study are available from the corresponding author (haruhisa@cira.kyoto-u.ac.jp) upon reasonable request.

## References

[1] Gandhi RT , Lynch JB and Del Rio C (2020) Mild or moderate Covid‐19. N Engl J Med 383, 1757–1766. 32329974 10.1056/NEJMcp2009249

[2] Viner RM , Russell SJ , Croker H , Packer J , Ward J , Stansfield C , Mytton O , Bonell C and Booy R (2020) School closure and management practices during coronavirus outbreaks including COVID‐19: a rapid systematic review. Lancet Child Adolesc Health 4, 397–404. 32272089 10.1016/S2352-4642(20)30095-X PMC7270629

[3] Wu F , Zhao S , Yu B , Chen Y‐M , Wang W , Song Z‐G , Hu Y , Tao Z‐W , Tian J‐H , Pei Y‐Y *et al*. (2020) A new coronavirus associated with human respiratory disease in China. Nature 579, 265–269. 32015508 10.1038/s41586-020-2008-3 PMC7094943

[4] Sperk M , van Domselaar R , Rodriguez JE , Mikaeloff F , Sá Vinhas B , Saccon E , Sönnerborg A , Singh K *et al*. (2020) Utility of proteomics in emerging and re‐emerging infectious diseases caused by RNA viruses. J Proteome Res 19, 4259–4274. 33095583 10.1021/acs.jproteome.0c00380 PMC7640957

[5] Kuroya M and Ishida N (1953) Newborn virus pneumonitis (type Sendai). II. The isolation of a new virus possessing hemagglutinin activity. Yokohama Med Bull 4, 217–233. 13137076

[6] Shioda T , Iwasaki K and Shibuta H (1986) Determination of the complete nucleotide sequence of the Sendai virus genome RNA and the predicted amino acid sequences of the F, HN and L proteins. Nucleic Acids Res 14, 1545–1563. 3005975 10.1093/nar/14.4.1545 PMC339528

[7] Takeuchi H , Imamura K , Ji B , Tsukita K , Enami T , Takao K , Miyakawa T , Hasegawa M , Sahara N , Iwata N *et al*. (2020) Nasal vaccine delivery attenuates brain pathology and cognitive impairment in tauopathy model mice. NPJ Vaccines 5, 28. 10.1038/s41541-020-0172-y PMC7096417 32219000

[8] Adderson E , Branum K , Sealy RE , Jones BG , Surman SL , Penkert R , Freiden P , Slobod KS , Gaur AH , Hayden RT *et al*. (2015) Safety and immunogenicity of an intranasal Sendai virus‐based human parainfluenza virus type 1 vaccine in 3‐ to 6‐year‐old children. Clin Vaccine Immunol 22, 298–303. 25552633 10.1128/CVI.00618-14 PMC4340902

[9] Yonemitsu Y , Matsumoto T , Itoh H , Okazaki J , Uchiyama M , Yoshida K , Onimaru M , Onohara T , Inoguchi H , Kyuragi R *et al*. (2013) DVC1‐0101 to treat peripheral arterial disease: a Phase I/IIa open‐label dose‐escalation clinical trial. Mol Ther 21, 707–714. 23319060 10.1038/mt.2012.279 PMC3589164

[10] Tai JA , Chang CY , Nishikawa T and Kaneda Y (2019) Cancer immunotherapy using the Fusion gene of Sendai virus. Cancer Gene Ther 27, 498–508. 31383952 10.1038/s41417-019-0126-6

[11] Shi Y , Inoue H , Wu JC and Yamanaka S (2017) Induced pluripotent stem cell technology: a decade of progress. Nat Rev Drug Discov 16, 115–130. 27980341 10.1038/nrd.2016.245 PMC6416143

[12] Imamura K , Izumi Y , Watanabe A , Tsukita K , Woltjen K , Yamamoto T , Hotta A , Kondo T , Kitaoka S , Ohta A *et al*. (2017) The Src/c‐Abl pathway is a potential therapeutic target in amyotrophic lateral sclerosis. Sci Transl Med 9, eaaf3962. 28539470 10.1126/scitranslmed.aaf3962

[13] Vershkov D , Fainstein N , Suissa S , Golan‐Lev T , Ben‐Hur T and Benvenisty N (2019) FMR1 reactivating treatments in fragile X iPSC‐derived neural progenitors *in vitro* and *in vivo* . Cell Rep 26, 2531–2539.e2534. 30840878 10.1016/j.celrep.2019.02.026

[14] Okita K , Yamakawa T , Matsumura Y , Sato Y , Amano N , Watanabe A , Goshima N and Yamanaka S (2013) An efficient nonviral method to generate integration‐free human‐induced pluripotent stem cells from cord blood and peripheral blood cells. Stem Cells 31, 458–466. 23193063 10.1002/stem.1293

[15] Hoenen T , Watt A , Mora A and Feldmann H (2014) Modeling the lifecycle of Ebola virus under biosafety level 2 conditions with virus‐like particles containing tetracistronic minigenomes. J Vis Exp 91, 52381. 10.3791/52381 PMC4828136 25285674

[16] Ianevski A , Giri AK and Aittokallio T (2020) SynergyFinder 2.0: visual analytics of multi‐drug combination synergies. Nucleic Acids Res 48, W488–W493. 32246720 10.1093/nar/gkaa216 PMC7319457

[17] Hormi M , Birsen R , Belhadj M , Huynh T , Cantero Aguilar L , Grignano E , Haddaoui L , Guillonneau F , Mayeux P , Hunault M *et al*. (2020) Pairing MCL‐1 inhibition with venetoclax improves therapeutic efficiency of BH3‐mimetics in AML. Eur J Haematol 105, 588–596. 32659848 10.1111/ejh.13492

[18] Li HO , Zhu Y‐F , Asakawa M , Kuma H , Hirata T , Ueda Y , Lee Y‐S , Fukumura M , Iida A , Kato A *et al*. (2000) A cytoplasmic RNA vector derived from nontransmissible Sendai virus with efficient gene transfer and expression. J Virol 74, 6564–6569. 10864670 10.1128/jvi.74.14.6564-6569.2000 PMC112166

[19] Warren TK , Jordan R , Lo MK , Ray AS , Mackman RL , Soloveva V , Siegel D , Perron M , Bannister R , Hui HC *et al*. (2016) Therapeutic efficacy of the small molecule GS‐5734 against Ebola virus in rhesus monkeys. Nature 531, 381–385. 26934220 10.1038/nature17180 PMC5551389

[20] Riva L , Yuan S , Yin X , Martin‐Sancho L , Matsunaga N , Pache L , Burgstaller‐Muehlbacher S , De Jesus PD , Teriete P , Hull MV *et al*. (2020) Discovery of SARS‐CoV‐2 antiviral drugs through large‐scale compound repurposing. Nature 586, 113–119. 32707573 10.1038/s41586-020-2577-1 PMC7603405

[21] Johansen LM , Brannan JM , Delos SE , Shoemaker CJ , Stossel A , Lear C , Hoffstrom BG , DeWald LE , Schornberg KL , Scully C *et al*. (2013) FDA‐approved selective estrogen receptor modulators inhibit Ebola virus infection. Sci Transl Med 5, 190ra179. 10.1126/scitranslmed.3005471 PMC3955358 23785035

[22] Kouznetsova J , Sun W , Martínez‐Romero C , Tawa G , Shinn P , Chen CZ , Schimmer A , Sanderson P , McKew JC , Zheng W and *et al*. (2014) Identification of 53 compounds that block Ebola virus‐like particle entry via a repurposing screen of approved drugs. Emerg Microbes Infect 3, e84. 26038505 10.1038/emi.2014.88 PMC4317638

[23] Hoenen T , Groseth A and Feldmann H (2019) Therapeutic strategies to target the Ebola virus life cycle. Nat Rev Microbiol 17, 593–606. 31341272 10.1038/s41579-019-0233-2

[24] Shiraki K and Daikoku T (2020) Favipiravir, an anti‐influenza drug against life‐threatening RNA virus infections. Pharmacol Ther 209, 107512. 32097670 10.1016/j.pharmthera.2020.107512 PMC7102570

[25] Tang YQ , Ye Q , Huang H and Zheng WY (2020) An overview of available antimalarials: discovery, mode of action and drug resistance. Curr Mol Med 20, 583–592. 32031068 10.2174/1566524020666200207123253

[26] Sarzi‐Puttini P , Ceribelli A , Marotto D , Batticciotto A and Atzeni F (2019) Systemic rheumatic diseases: from biological agents to small molecules. Autoimmun Rev 18, 583–592. 30959214 10.1016/j.autrev.2018.12.009

[27] Gordon DE , Jang GM , Bouhaddou M , Xu J , Obernier K , White KM , O'Meara MJ , Rezelj VV , Guo JZ , Swaney DL *et al*. (2020) A SARS‐CoV‐2 protein interaction map reveals targets for drug repurposing. Nature 583, 459–468. 32353859 10.1038/s41586-020-2286-9 PMC7431030

[28] Sacco MD , Ma C , Lagarias P , Gao A , Townsend JA , Meng X , Dube P , Zhang X , Hu Y , Kitamura N *et al*. (2020) Structure and inhibition of the SARS‐CoV‐2 main protease reveal strategy for developing dual inhibitors against M(pro) and cathepsin L. Sci Adv 6, eabe0751. 33158912 10.1126/sciadv.abe0751 PMC7725459

[29] Vuong W , Khan MB , Fischer C , Arutyunova E , Lamer T , Shields J , Saffran HA , McKay RT , van Belkum MJ , Joyce MA *et al*. (2020) Feline coronavirus drug inhibits the main protease of SARS‐CoV‐2 and blocks virus replication. Nat Commun 11, 4282. 32855413 10.1038/s41467-020-18096-2 PMC7453019

[30] Ma C , Sacco MD , Hurst B , Townsend JA , Hu Y , Szeto T , Zhang X , Tarbet B , Marty MT , Chen Y and *et al*. (2020) Boceprevir, GC‐376, and calpain inhibitors II, XII inhibit SARS‐CoV‐2 viral replication by targeting the viral main protease. Cell Res 30, 678–692. 32541865 10.1038/s41422-020-0356-z PMC7294525

[31] Shin D , Mukherjee R , Grewe D , Bojkova D , Baek K , Bhattacharya A , Schulz L , Widera M , Mehdipour AR , Tascher G *et al*. (2020) Papain‐like protease regulates SARS‐CoV‐2 viral spread and innate immunity. Nature 587, 657–662. 32726803 10.1038/s41586-020-2601-5 PMC7116779

[32] Rut W , Groborz K , Zhang L , Sun X , Zmudzinski M , Pawlik B , Wang X , Jochmans D , Neyts J , Młynarski W *et al*. (2020) Activity profiling and crystal structures of inhibitor‐bound SARS‐CoV‐2 papain‐like protease: a framework for anti‐COVID‐19 drug design. Sci Adv 6, eabd4596. 33067239 10.1126/sciadv.abd4596 PMC7567588

[33] Galindo I , Garaigorta U , Lasala F , Cuesta‐Geijo MA , Bueno P , Gil C , Delgado R , Gastaminza P and Alonso C (2020) Antiviral drugs targeting endosomal membrane proteins inhibit distant animal and human pathogenic viruses. Antiviral Res 186, 104990. 33249093 10.1016/j.antiviral.2020.104990 PMC7690281

[34] Jeon S , Ko M , Lee J , Choi I , Byun SY , Park S , Shum D and Kim S (2020) Identification of antiviral drug candidates against SARS‐CoV‐2 from FDA‐approved drugs. Antimicrob Agents Chemother 64, e00819‐20. 32366720 10.1128/AAC.00819-20 PMC7318052

[35] Weston S , Coleman CM , Haupt R , Logue J , Matthews K , Li Y , Reyes HM , Weiss SR and Frieman MB (2020) Broad anti‐coronavirus activity of food and drug administration‐approved drugs against SARS‐CoV‐2 *in vitro* and SARS‐CoV *in vivo* . J Virol 94, e01218‐20. 32817221 10.1128/JVI.01218-20 PMC7565640

[36] Eyre NS , Kirby EN , Anfiteatro DR , Bracho G , Russo AG , White PA , Aloia AL and Beard MR (2020) Identification of estrogen receptor modulators as inhibitors of flavivirus infection. Antimicrob Agents Chemother 64, e00289‐20. 32482672 10.1128/AAC.00289-20 PMC7526829

[37] Fan H , Du X , Zhang J , Zheng H , Lu X , Wu Q , Li H , Wang H , Shi Y , Gao G *et al*. (2017) Selective inhibition of Ebola entry with selective estrogen receptor modulators by disrupting the endolysosomal calcium. Sci Rep 7, 41226. 28117364 10.1038/srep41226 PMC5259750

[38] Zhou Y , Hou Y , Shen J , Huang Y , Martin W and Cheng F (2020) Network‐based drug repurposing for novel coronavirus 2019‐nCoV/SARS‐CoV‐2. Cell Discov 6, 14. 10.1038/s41421-020-0153-3 PMC7073332 32194980

[39] Behjati S and Frank MH (2009) The effects of tamoxifen on immunity. Curr Med Chem 16, 3076–3080. 19689284 10.2174/092986709788803042 PMC2902982

[40] Cerciat M , Unkila M , Garcia‐Segura LM and Arevalo MA (2010) Selective estrogen receptor modulators decrease the production of interleukin‐6 and interferon‐gamma‐inducible protein‐10 by astrocytes exposed to inflammatory challenge *in vitro* . Glia 58, 93–102. 19533603 10.1002/glia.20904

[41] Suuronen T , Nuutinen T , Huuskonen J , Ojala J , Thornell A and Salminen A (2005) Anti‐inflammatory effect of selective estrogen receptor modulators (SERMs) in microglial cells. Inflamm Res 54, 194–203. 15953991 10.1007/s00011-005-1343-z

[42] Azizian H , Khaksari M , Asadikaram G , Sepehri G and Najafipour H (2018) Therapeutic effects of tamoxifen on metabolic parameters and cytokines modulation in rat model of postmenopausal diabetic cardiovascular dysfunction: role of classic estrogen receptors. Int Immunopharmacol 65, 190–198. 30316077 10.1016/j.intimp.2018.10.009

[43] Smetana K Jr , Rosel D and Br Ábek J (2020) Raloxifene and bazedoxifene could be promising candidates for preventing the COVID‐19 related cytokine storm, ARDS and mortality. In Vivo 34, 3027–3028. 32871847 10.21873/invivo.12135 PMC7652447

[44] Darwish I , Mubareka S and Liles WC (2011) Immunomodulatory therapy for severe influenza. Expert Rev Anti Infect Ther 9, 807–822. 21810053 10.1586/eri.11.56

[45] Omeragic A , Kara‐Yacoubian N , Kelschenbach J , Sahin C , Cummins CL , Volsky DJ and Bendayan R (2019) Peroxisome proliferator‐activated receptor‐gamma agonists exhibit anti‐inflammatory and antiviral effects in an EcoHIV mouse model. Sci Rep 9, 9428. 31263138 10.1038/s41598-019-45878-6 PMC6603270

[46] Luzi L and Radaelli MG (2020) Influenza and obesity: its odd relationship and the lessons for COVID‐19 pandemic. Acta Diabetol 57, 759–764. 32249357 10.1007/s00592-020-01522-8 PMC7130453

[47] Takayama K (2020) *In vitro* and animal models for SARS‐CoV‐2 research. Trends Pharmacol Sci 41, 513–517. 32553545 10.1016/j.tips.2020.05.005 PMC7260555

[48] Matsuyama S , Nao N , Shirato K , Kawase M , Saito S , Takayama I , Nagata N , Sekizuka T , Katoh H , Kato F *et al*. (2020) Enhanced isolation of SARS‐CoV‐2 by TMPRSS2‐expressing cells. Proc Natl Acad Sci USA 117, 7001–7003. 32165541 10.1073/pnas.2002589117 PMC7132130

[49] Leist SR , Schäfer A and Martinez DR (2020) Cell and animal models of SARS‐CoV‐2 pathogenesis and immunity. Dis Model Mech 13, dmm046581. 32887790 10.1242/dmm.046581 PMC7490513

[50] Hekman RM , Hume AJ , Goel RK , Abo KM , Huang J , Blum BC , Werder RB , Suder EL , Paul I , Phanse S *et al*. (2020) Actionable cytopathogenic host responses of human alveolar type 2 cells to SARS‐CoV‐2. Mol Cell 80, 1104–1122.e1109. 33259812 10.1016/j.molcel.2020.11.028 PMC7674017

