# Supplementary material for: iPSC screening for drug repurposing identifies anti‐RNA virus agents modulating host cell susceptibility
Source: FEBS Open Bio. 2021 Apr 6;11(5):1452–64. doi: 10.1002/2211-5463.13153 (PMC8091584; doi:10.1002/2211-5463.13153)
Supplement: Supplementary file 1 — Fig. S1. Karyotype analysis of iPSCs and evaluation of an RNA‐dependent RNA polymerase inhibitor. A. Karyotype analysis of iPSCs. B. Quantification of viral mRNA levels. Pre‐treatment of 1 μm Remdesivir significantly decreased EGFP mRNA levels (n = 3; student t‐test, * P < 0.005). Bar graphs represent mean ± SEM. Fig. S2. Antiviral activity of SERMs against Ebola trVLP. Dose‐response analysis of SERMs against the infectivity of Ebola trVLP. Tamoxifene, Toremifene, and Clomifene inhibited Ebola trVLP infection showing infectivity (blue) and cell survival (green). Data are normalized to mean values for untreated control and presented as mean ± SEM. n = 3, biological replicates. Table S1. Primer list for qPCR. [file FEB4-11-1452-s001.pdf]

Supplementary Materials:

Figure S1. Karyotype analysis of iPSCs and evaluation of an RNA-dependent RNA polymerase inhibitor

Figure S2. Anti-viral activity of SERMs against Ebola trVLP

Table S1. Primer list for qPCR

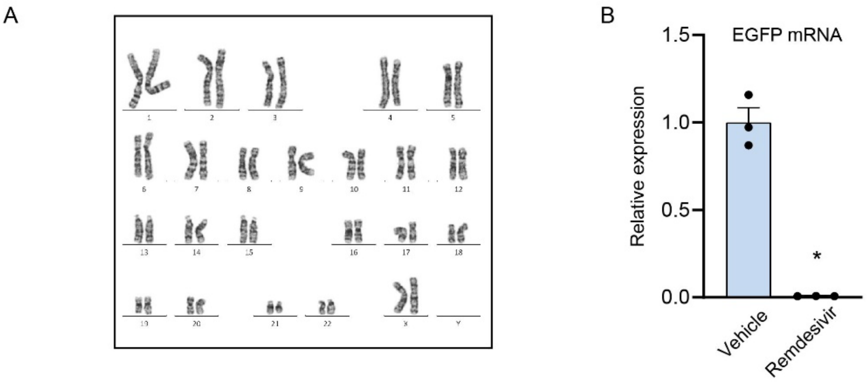

Figure S1. Karyotype analysis of iPSCs and evaluation of an RNA-dependent RNA polymerase inhibitor

A. Karyotype analysis of iPSCs.

B. Quantification of viral mRNA levels. Pre-treatment of 1 μM Remdesivir significantly decreased EGFP mRNA levels (n = 3; student *t*-test, * $p < 0.005$). Bar graphs represent mean ± SEM.

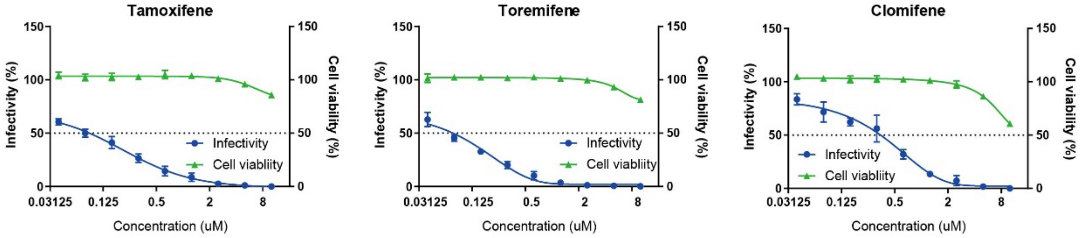

Figure S2. Anti-viral activity of SERMs against Ebola trVLP

Dose-response analysis of SERMs against the infectivity of Ebola trVLP. Tamoxifene, Toremifene, and Clomifene inhibited Ebola trVLP infection showing infectivity (blue) and cell survival (green). Data are normalized to mean values for untreated control and presented as mean ± SEM. n = 3, biological replicates.

Table S1. Primer list for qPCR

Primer list	Sequences (5' to 3')
EGFP_F	GGACGACGGCAACTACAAGA
EGFP_R	TTGTACTCCAGCTTGTGCCC
GAPDH_F	TCCACTGGCGTCTTCACC
GAPDH_R	GGCAGAGATGATGACCCTTTT
